# Treatment time and learning curve analysis of 1.5 T MR-Linac workflows led by radiation oncologists or therapists

**DOI:** 10.1016/j.ctro.2024.100901

**Published:** 2024-12-14

**Authors:** J.M. Westerhoff, F.J. Raaijmakers, L.A. Daamen, E.N. de Groot-van Breugel, L.T.C. Meijers, J.R.N. van der Voort van Zyp, J.J.C. Verhoeff, S. Mook, H.M. Verkooijen, M.P.W. Intven

**Affiliations:** a University Medical Center Utrecht, Division of Imaging and Oncology, Utrecht, the Netherlands; b University Medical Center Utrecht, Department of Radiation Oncology, Utrecht, the Netherlands

## Abstract

**Background and purpose:**

This study assessed the treatment time of online adaptive (i.e. Adapt-to-Shape, ATS) and virtual couch shift (i.e. Adapt-to-Position, ATP) magnetic resonance guided radiotherapy (MRgRT) on a 1.5 Tesla MR-Linac. Additionally, the transition from a radiation oncologists (RO)-led to a radiation therapist (RTT)-led workflow, and the presence of a learning curve were assessed.

**Materials and methods:**

This study was conducted utilizing the prospective Multi-OutcoMe EvaluatioN of radiation Therapy Using the MR-Linac study (MOMENTUM, NCT04075305). Mean (±standard deviation) online adaptation time and total treatment time were collected from MR-Linac log files. Learning and proficiency phases were defined using a cumulative sum (CUSUM) analysis. Independent T-tests were performed. A p-value < 0.01 was considered statistically significant.

**Results:**

In total, 4942 fractions of 645 patients were included. Mean total treatment time was 39 (±7), 15 (±2), 34 (±8), 41 (±11), and 40 (±7) minutes for ATS-treated prostate cancer, ATP-treated prostate cancer, ATS-treated pelvic lymph node metstasis (LNM), ATS-treated abdominal LNM and ATS-treated rectal cancer, respectively. Mean online adaptation time of RO-led and RTT-led treatment was 28 (±6) and 25 (±6) minutes (p < 0.001) for ATS-treated prostate cancer. No significant differences in the remaining subgroups were found. In subgroups with a learning curve, mean online adaptation time of learning and proficiency phase were 30 (±6) and 26 (±5) minutes (p < 0.001) for ATS-treated prostate cancer, 27 (±8) and 19 (±7) minutes for pelvic LNM (p < 0.001), and 29 (±7) and 25 (±7) minutes (p < 0.001) for rectal cancer, respectively.

**Conclusion:**

The transition from RO-led to RTT-led workflows did not significantly increase total treatment time. The online adaptation time reduced after a learning curve for ATS-treated prostate cancer, pelvic LNM and rectal cancer.

**Keywords**; MRgRT, MR-Linac, time, prostate cancer, oligolymphnode metastasis, rectal cancer.

## Highlights

• Online adaptive magnetic resonance guided radiotherapy (MRgRT) was delivered within 45 minutes in 73%–83%, depending on subgroup.
• The transition from radiation oncologist (RO) led to radiation therapists (RTT) led workflow did not significantly increase treatment time.
• A learning curve of treatment time was found for RTT-led online adaptive MRgRT of prostate cancer, pelvic lymph node metastasis and rectal cancer.
• The found learning curve significantly reduced treatment time with four to eight minutes.

## Introduction

Magnetic resonance guided radiation therapy (MRgRT) aims to improve the accuracy of radiotherapy with superior soft tissue contrast of magnetic resonance imaging (MRI). Together with the available online adaptive workflow, Adapt-to-Shape (ATS), a new on-table treatment plan can be generated to match the anatomy of the day [1]. The improved accuracy could enable hypofractionation or a reduction of treatment margins, which could lead to reduced toxicity, and allow for dose escalation, potentially resulting in improved local tumor control. However, MRI acquisition and online adaptive workflow increase treatment duration, one of the concerns when introducing online adaptive MRgRT. Treatment times of computed tomography (CT)-guided radiotherapy (CTgRT) ranged from 8 to 10 min, while early safety and feasibility studies report treatment times between 45 and 60 min for MRgRT [2], [3], [4], [5], [6].

Increased treatment times impact the precision of radiotherapy, patient experience, complicate hospital logistics, and require additional human and financial resources [7], [8], [9]. With increased treatment time, the risk of intrafraction anatomical motion increases [10]. For example, during online adaptation of prostate cancer MRgRT, the bladder continues to fill, and pelvic muscles relax. Intrafraction motion during the adaptation phase can be resolved by additional position verification and second plan adaptation. However, this further increases treatment time. During beam-on time, intrafraction motion can be accounted for with gating or tracking. This also increases treatment time when targets move substantially, and is currently not yet widely available among the 1.5 Tesla (T) MR-Linac devices.

Secondly, treatment time impacts patient experience [11]. This is particularly relevant considering that MRgRT takes place within a confined bore with noise from the MRI. Although music or video is available to make the treatment more comfortable.

Finally, the treatment time impacts available resources and subsequently the potential for the treatment to become cost-effective. A treatment fraction is more costly when delivered with MRgRT compared to conventional devices [12], [13]. A fair amount of these costs arise from medical staff available during treatment. In certain countries, radiation therapists (RTTs) are authorized to plan and deliver treatments, whereas in other countries the presence of an extra physician or physicist is required. A reduction of treatment time will decrease the costs of treatment, especially in countries where the presence of a physician or physicist is required. A switch to an RTT-led workflow would be a possibility to increase efficiency, if this does not increase treatment time.

The aim of this study was to report treatment times, investigate the presence of an institutional learning curve, and evaluate the impact of transitioning to an RTT-led workflow on treatment time.

## Materials and methods

### Study population

This study was conducted within the prospective Multi-OutcoMe EvaluatioN of radiation Therapy Using the MR-linac study (MOMENTUM, NCT04075305) which received approval from the local institution review board [14]. Starting in 2019, MOMENTUM has been enrolling patients over 18 years old who receive treatment on the 1.5 T MR-Linac (Unity, Elekta AB, Stockholm, Sweden) and provide written informed consent. Included were patients treated according to standard practices in the University Medical Center (UMC) Utrecht between November 2018 and November 2023. Selected were subgroups of patients with over 200 delivered treatment fractions. Excluded were patients that participated in an interventional study.

### Treatment

**Fig. 1:**
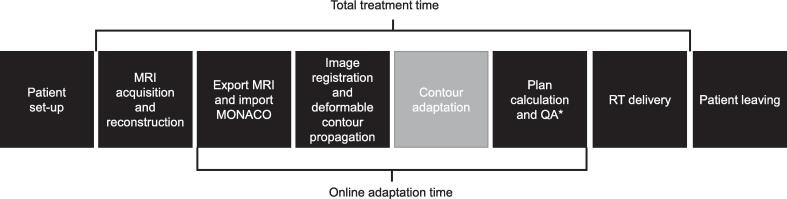
Worfklow for treatment on the MR-Linac with definition of total treatment time and online adaptation time. For the ATS workflow, all steps are performed for each treatment fraction. For the ATP workflow, only the black steps are performed at each treatment fraction. *Plan calculation and QA is a step in both ATS and ATP workflows. However, the plan calculation and QA comprises of a full optimalization in the ATS workflow, while this is less elaborate in the ATP workflow. Abbreviations; ATS, Adapt-to-Shape; ATP, Adapt-to-Position; MRI, magnetic resonance imaging; QA, quality assurance; RT, radiotherapy.

The UMC Utrecht started treating patients on the MR-Linac in August 2018. Initially, only the ATS workflow was used to treat patients, where a new plan is generated after manual adaptation of delineations from targets and organs at risk (OARs) within 2 cm proximity of the target. All steps of the treatment workflow are presented in Fig. 1 and were published previously [1], [15]. Daily planning aimed to adhere to all clinical objectives for every fraction. It was accepted to violate a clinical objective when the original pre-plan did too. In February 2021, the Adapt-to-Position (ATP) workflow, which is a virtual couch shift, was introduced for limited high risk prostate cancer. Patients were generally treated using one workflow. Only in few cases when intrafraction motion during the first workflow caused impaired plan quality, a new ATP workflow was performed.

*RO-led treatment*.

At first, the treatment team comprised three RTTs and a radiation oncologist (RO). A physicist was available on call. ROs generated and approved plans, and were supported by RTTs. After this, RTTs were gradually trained to perform the steps individually under supervision of ROs. All of which is defined as RO-led.

*RTT-led treatment*.

The new RTT-led workflow was adopted for lymph node metastasis (LNM) in December 2019, and for prostate and rectal cancer in January 2020. All included ATP treatments were led by RTTs. The RTT-led workflow was performed by three RTTs. The responsibilities were divided as follows; (1) patient communication and operation of the MR console, (2) image processing and contouring, (3) treatment planning and plan QA. A physicist and RO were available on call and were consulted when needed or in case of violation of a new clinical objective. A traffic light system quickly evaluated whether the daily plan varied from the original plan. ROs reviewed plans offline. The total team of MR-Linac specialized RTTs started with 4 and expanded to 20. New members were trained one at a time. The structure of the RTT-training with learning objectives can be found in Supplemental Fig. 1.

### Study outcomes and data collection

Outcomes of interest were online adaptation time and total treatment time (Fig. 1). Online adaptation time was measured from the end of the first MRI until the moment that the plan was approved and exported to be delivered. Online adaptation time included registration of images, deformable contour propagation, manual adaptation of delineations, full plan optimization, and quality assurance (QA). Total treatment time was measured from the start of the first MRI until the end of the radiotherapy delivery. The following time points were captured from log files of the MR-Linac; start of first MRI, plan ready, end of radiotherapy delivery. The MRI duration was determined based on used MRI sequence, which were both in-house and manufacturer developed.

### Analysis

Analyses were performed for five subgroups; ATP-treated prostate cancer, ATS-treated prostate cancer, ATS-treated pelvic LNM, ATS-treated abdominal LNM, and ATS-treated rectal cancer. Online adaptation time and total treatment time were presented using mean ± standard deviation (SD). Medical records of patients with treatment durations within the upper 2.5th percentile were reviewed. Excluded were treatment fractions with a technical error, or with a discontinuation of treatment. Different phases of learning were identified using a cumulative sum (CUSUM) analysis [16]. For this analysis, all RTT-led fractions were consecutively ranked based on date, and the difference to the mean online adaptation time of the specific subgroup for each treatment fraction was calculated. The cumulative sum of these differences was plotted. An upward slope represents a phase where treatment time is above the mean. A downward slope represents a phase where treatment time is below the mean. The steeper the slope, the larger the difference from the mean. The inflection point from an upward to a downward slope represents the transition from a learning phase to a proficiency phase. A learning curve was defined as a CUSUM analysis starting with an upward slope, followed by a downward slope. No CUSUM analysis was performed for RO-led treatment fractions, as the MOMENTUM study was initiated after the clinical introduction of the MR-Linac and data of the first fractions is missing. The inflection point of a CUSUM analysis largely depends on the amount of available data per subgroup [17]. Consequently, it was preferred to determine the inflection point of RTT-led subgroups of equal size. On the other hand, excluding large proportions of available data was considered undesirable. Therefore, It was decided to first utilize all available data to identify whether a learning curve was present with a first CUSUM analysis. The number of included treatment fractions per subgroup with a learning curve was matched to the subgroup with the least available data. This was done by excluding the most recent treatment fractions. A second CUSUM analysis with this data was conducted to determine inflection points and subsequent learning and proficiency phases. Independent two-sided T-tests were conducted to test for significance. To reduce the risk of false positive results due to multiple testing, a p-value < 0.01 was considered statistically significant. All analysis were performed using R version 2023.10.

## Results

### Patients

Data of 676 patients with 5009 treatment fractions were collected. Excluded were 67 treatment fractions (Supplemental Fig. 2) as treatment was discontinued. This resulted in 4942 fractions of 645 patients (Table 1).

**Fig. 2:**
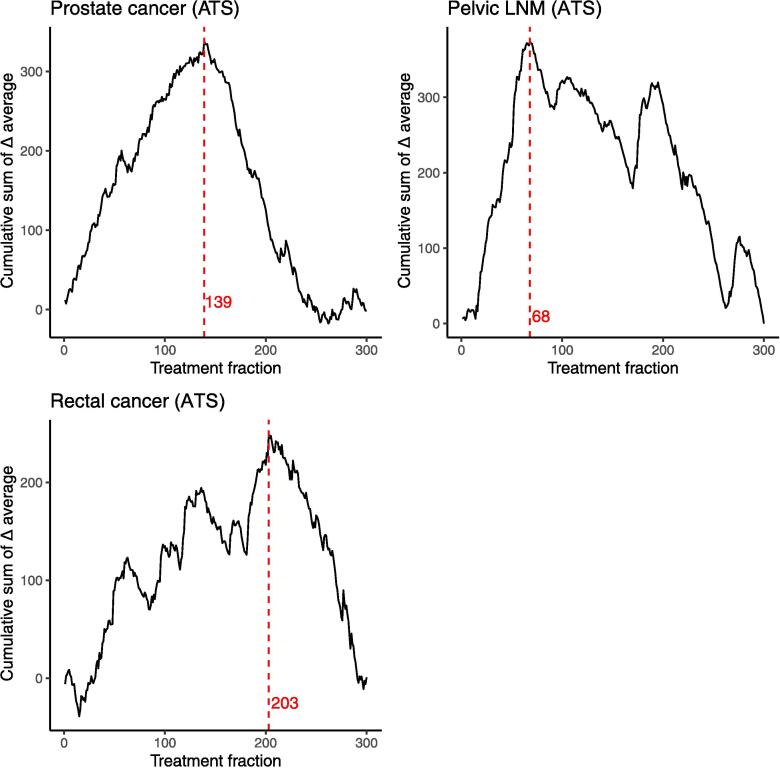
CUSUM analysis of RTT-led treatment fractions with a learning curve. The inflection point with subsequent sequence number are depicted in red, which represents the transition from learning phase to proficiency phase. Abbreviations; CUSUM, cumulative sum; RTT, radiation therapist; ATS, Adapt-to-Shape; LNM, lymph node metastasis. (For interpretation of the references to colour in this figure legend, the reader is referred to the web version of this article.)

**Table 1:** Baseline characteristics of the study population.

	**Prostate cancer (ATP)**	**Prostate cancer (ATS)**	**Pelvic LNM (ATS)**	**Abdominal LNM (ATS)**	**Rectal cancer (ATS)**
**Patients,** n	118	287	111	37	92
**Age,** median (range)	75 (59–84)	70 (51–85)	72 (46–87)	71 (40–83)	63 (37–84)
**Male gender,** n (%)	118 (100)	287 (100)	108 (97)	29 (78)	68 (74)
**Performance score,** **ECOG/ KPSS,** n (%)					
0 / 90–100	57 (48)	164 (57)	49 (44)	18 (49)	61 (66)
1 / 70–80	6 (5)	16 (6)	6 (5)	4 (11)	8 (9)
2 / 50–60	0 (0)	3 (1)	1 (1)	2 (5)	0 (0)
Not assessed	55 (47)	104 (36)	55 (50)	13 (35)	23 (25)
**Treatment fractions,** n	2316	1410	541	220	455
**Dose per fraction in Gy,** (median, range*)	3.1	7.25	7 (7–10)	7 (7–10)	5
**RTT-led treatments,** n (%)	2316 (100)	1320 (94)	407 (75)	160 (73)	318 (70)
Abbreviations; ATS, Adapt-to-Shape; ATP, Adapt-to-Position; LNM, Lymph Node Metastasis; ECOG, Eastern Cooperative Oncology Group; KPSS, Karnofsky Performance Status Scale; Gy, Gray; RTT, radiation therapist *Range was only presented when treatment dose varied within the subgroup					

### Treatment times

Mean total treatment time for prostate cancer was 15 (±2) minutes for ATP workflow and 39 (±7) minutes for the ATS workflow (Table 2). Mean total treatment time was 34 (±8) minutes for pelvic LNM, 41 (±11) minutes for abdominal LNM, and 40 (±7) minutes for rectal cancer. For prostate cancer treated with ATP, 100 % (n = 2315) of fractions were delivered within 30 min and 68 % (n = 1580) within 15 min. For all ATS-treatments, 95 % to 99 % were delivered within 60 min, and 73 % to 89 % were delivered within 45 min, depending on subgroup. For RO-led treatment an upward trend was found for pelvic LNM, abdominal LNM, and rectal cancer (Supplemental Fig. 3). For RTT-led treatments a downward trend was found for all ATS-treated subgroups, except for abdominal LNM.

**Table 2:** Mean total treatment times and duration of different phases of a treatment fraction.

	**MRI acquisition (min), mean (SD)**	**Online adaptation time (min), mean (SD)**	**RT delivery (min), mean (SD)**	**Total treatment time (min), mean (SD)**	**Total treatment time < 60 min, n (%)**	**Total treatment time < 45 min, n (%)**
Prostate cancer (ATP)	3 (0.3)	5 (2)	7 (1)	15 (2)	2316 (100)	2315 (100)
Prostate cancer (ATS)	3 (1)	26 (6)	11 (2)	39 (7)	1392 (99)	1196 (85)
Pelvic LNM (ATS)	3 (1)	21 (7)	10 (3)	34 (8)	486 (99)	436 (89)
Abdominal LNM (ATS)	4 (1)	25 (8)	12 (6)	41 (11)	158 (95)	122 (73)
Rectal cancer (ATS)	4 (1)	27 (7)	9 (2)	40 (7)	443 (97)	368 (81)
Abbreviations; ATS, Adapt-to-Shape; ATP, Adapt-to-Position; LNM, Lymph Node Metastasis; min, minutes; MRI, magnetic resonance imaging; RT, radiotherapy; SD, standard deviation						

### RO-led versus RTT-led workflow

A significant reduction from RO-led to RTT-led mean online adaptation time in minutes was found for ATS-treated prostate cancer (28 (±6) versus 25 (±6), p < 0.001) (Table 3). No significant change in online adaptation time in minutes was found for pelvic LNM (21 (±7) versus 21 (±7), p = 0.778), abdominal LNM (23 (±7) versus 25 (±8), p = 0.094) and rectal cancer (27 (±6) versus 27 (±7), p = 0.998).

**Table 3:** Treatment times of RO-led versus RTT-led treatment fractions.

	**Online adaptation time (min), mean (SD)**			**Total treatment time (min), mean (SD)**		
	**RO-led**	**RTT-led**	p-value*	**RO-led**	**RTT-led**	p-value*
**Prostate cancer (ATS)**	28 (6)	25 (6)	<0.001	41 (7)	39 (7)	0.021
**Pelvic LNM (ATS)**	21 (7)	21 (7)	0.778	33 (7)	34 (8)	0.103
**Abdominal LNM (ATS)**	23 (7)	25 (8)	0.094	38 (10)	40 (10)	0.113
**Rectal cancer (ATS)**	27 (6)	27 (7)	0.998	39 (7)	41 (8)	0.013
Abbreviations; RO, radiation oncologist; RTT, radiation therapist; ATS, Adapt-to-Shape; SD, standard deviation; min, minutes *Independent T-Test						

### Learning curve

Based on all available data, a learning curve was found for ATS-treated prostate cancer, pelvic LNM and rectal cancer (Supplemental Fig. 4). No learning curve was found for ATP-treated prostate cancer and abdominal LNM. Based on the first 300 available RTT-led treatment fractions, inflection points from learning to proficiency phases were found at fraction 139, 68, and 203 for ATS-treated prostate cancer, pelvic LNM, and rectal cancer, respectively (Fig. 2). Between learning and proficiency phase, a significant reduction in online adaptation time and total treatment time in minutes was found for ATS-treated prostate cancer (30 (±6) versus 26 (±5), p < 0.001, and 43 (±6) versus 39 (±6), p < 0.001) and pelvic LNM (27 (±8) versus 19 (±7), p < 0.001 and 40 (±8) versus 33 (±8), p < 0.001) (Table 4). For rectal cancer, a significant reduction between learning and proficiency phase was only found for the online adaption time (29 (±7) versus 25 (±7), p < 0.001), as radiotherapy delivery time increased over time (Supplemental Fig. 5).

**Table 4:** Mean online adaptation times and total treatment times of the learning and the proficiency phases.

		**Online adaptation time (min), mean (SD)**			**Total treatment time (min), mean (SD)**		
	Inflection point*, fraction	**Learning**	**Proficiency**	p-value**	**Learning**	**Proficiency**	p-value**
**Prostate cancer (ATS)**	139	30 (6)	26 (5)	<0.001	43 (6)	39 (6)	<0.001
**Pelvic LNM (ATS)**	68	27 (8)	19 (7)	<0.001	40 (8)	33 (8)	<0.001
**Rectal cancer (ATS)**	203	29 (7)	25 (7)	<0.001	41 (7)	40 (8)	0.194
Abbreviations; ATS, Adapt-to-Shape; min, minutes; SD, standard deviation; RTT, radiation therapists; LNM, lymph node metastasis *Inflection point based on the CUSUM analysis of online adaptation time from the first 300 cases that were RTT led. This point reflects the transition from learning phase to proficiency phase. **Independent T-test							

## Discussion

We found a mean treatment time for the ATS workflow of 34 to 41 min, depending on treatment site. Mean treatment time for the ATP workflow for prostate cancer was 15 min. The transition from an RO to an RTT-led workflow did not significantly increase treatment time. Furthermore, we found a learning curve in ATS workflows led by RTTs for prostate cancer, pelvic LNM, and rectal cancer, reducing the online adaptation time with four to eight minutes.

Since the introduction of online adaptive MRgRT, concerns were raised regarding the additional time and resources needed. We present the treatment time of a large cohort of patients, with the advantage that operators were not aware of the timing being evaluated. These results are important to estimate the required resources, support the estimation of cost-effectiveness and to provide a benchmark for future development.

The found mean treatment time of 39 min for ATS-treated prostate cancer is in line with literature [[4], [6]]. The shorter ATP-treatment for prostate cancer with a mean of 15 min can partly be explained by the lower dose of ATP treatment fractions as compared to ATS (3.1 Gy versus 7.25 Gy). However, the largest time saving rises from the fact that no online adaptation was performed. Despite the increased treatment duration, ATS is the workflow of choice for the hypofractionated treatment of the included subgroups, as it is believed that the precision increases with the use of ATS. We found a mean treatment time of 34 min for pelvic LNM and 41 min for abdominal LNM. This is notably shorter than the reported 58 to 66 min [18], [19]. This could be due to the use of gating by Kutuk et al. and Yang et al. while this study did not. Additionally, Kutuk et al. included patients with oligometastasis in the lung (18.5 %), liver (16.7 %), adrenal glands (11.1 %), and soft tissue (5.6 %). Interestingly, we found that the time for RT delivery in rectal cancer patients increased over time, which diminished the learning curve effect on total treatment time. This finding was attributed to the alteration of the treatment plan template around the same period, which increased the number of beams and segments per treatment fraction.

An important finding is that RTTs are equally efficient in terms of treatment time for the included subgroups as compared to ROs. Apart from efficiency, plan quality is likely even more important. Delineations of RTTs and ROs for prostate, rectal and LNM were previously compared in our institute [20], [21]. Both studies concluded that delineations were comparable, and switch to an RTT-led workflow was considered to be safe. With the finding that an RTT-led workflow did not significantly increase treatment time compared to an RO-led workflow, it can also considered to be efficient. Although safe and efficient, we acknowledge that in some countries legislation might be a barrier to this implementation.

Many of the new responsibilities of the ATS workflow for MRgRT also apply to online adaptive CTgRT. Similarly, there is an interest to switch to an RTT-led online adaptive workflow [22], [23]. This is described as feasible. Beckert et al. showed that training of RTTs decreases contouring time from 20 to 8 min [24]. However, the required RO review largely diminished this gain. This emphasizes not only the need for well-established training programs, but also for an independent RTT-led workflow.

The assessment of a learning curve in the field of radiotherapy is uncommon and limited to the delineation of a selective number of targets or OARs [25], [26], [27], [28], while this is common in the assessment of surgical procedures [16]. As the online adaptive radiotherapy workflow contains a substantial manual phase, a learning curve becomes of interest. As opposed to surgery, radiotherapy consists of a treatment course with multiple fractions delivered to a single patient. In the choice of analysis, we considered this possible underlying correlation of multiple treatment fractions per patient. However, since treatment is adapted to match the anatomy of a new daily MRI, the adjustments vary per treatment fraction. Moreover, in our large treatment team of RTTs, responsibilities switch regularly, so the chance of RTTs adapting subsequent treatments of the same patient is limited. This is why it was decided to consider each treatment fraction as independent. The CUSUM analysis enables the visualization of trends over time. The found learning curve for online adaptive treatment of prostate cancer, pelvic LNM and rectal cancer could justify the centralization of care in order to maximize efficiency. The interpretation of the inflection point should be done with care in a CUSUM analysis based on a sample mean. Lin et al. demonstrated that the inflection point is affected by the total number of included cases, caused by a change in sample mean [17]. The comparison of the inflection points can suggest which indications reach proficiency fastest, which was pelvic LNM. However, the absolute value of the inflection point is not a robust and consistent measure.

Notably, a learning curve was not found for all subgroups. We hypothesize that a learning curve was not found in our population of abdominal LNM due to heterogeneity of interfraction motion, number of OARs, relation of the target with surrounding OARs, or visibility of the target and OARs. Large variation in level of delineation difficulty could have a larger impact on duration than operators’ experience. The absence of a learning curve in prostate ATP is thought to be due to the minimal manual procedures during this workflow. Additionally, we found local valleys in the learning curve of pelvic LNM and rectal cancer, which reflect outliers with increased online adaptation time. These could be due to tumor-related factors (i.e. interfraction or intrafraction motion, poor visibility, number of OARs, relation of the target to surrounding OARs, challenging constraints) or staff related factors (i.e. training of new staff, waiting for physicist or RO in case of required support).

The findings of the present study should be interpreted in the light of several limitations. First, the MOMENTUM study was initiated after the clinical introduction of the MR-Linac. Consequently, the first cases included do not represent our institutes’ first. This is why a learning curve for ROs could not be estimated. Second, there were likely factors that influenced the learning curve and inflection points which we were unable to correct for. For example, patient acquisition rate, the treatment team size, previous MRI experience and training of staff. The inclusion of more difficult cases as experience grows could underestimate the found learning curve effect. The influence of training of new colleagues probably influences treatment time, however also reflects a real-world situation, for which we argue that correction is unnecessary. Finally, the patient set-up and leaving is not included in the reported total treatment time. As we believe this time to be generally equal over time, the learning curve analysis is not expected to be influenced. The patient set-up was estimated to be 6 to 7 min [3], [4], which should be taken into account while interpreting the total treatment time.

The future application of gating will likely increase the radiotherapy delivery time for targets with motion, which increases treatment time. We showed that institutional experience can contribute to the reduction of treatment time. Another promising strategy to improve efficiency is the application of artificial intelligence (AI) for contouring, planning, and image reconstruction [29], [30]. Also, RT delivery efficiency could be optimized by less treatment segments, or by implementation of volumetric-modulated arc therapy (VMAT). As we expect many future developments that will impact treatment time, it will be important to monitor it, so efficiency can be taken into account for the future development of MRgRT.

The analysis of our large prospective cohort study shows that online adaptive MRgRT of prostate cancer, pelvic LNM, abdominal LNM, and rectal cancer can generally be delivered within 45 min and all ATP treatment fractions of prostate cancer within 30 min. The switch from an RO to an RTT-led workflow did not significantly increase treatment duration. A learning curve was found for the online adaptive workflow of prostate cancer, pelvic LNM and rectal cancer, significantly reducing treatment time with four to eight minutes as experience increased. The learning curve effect moderately increased the efficiency of MRgRT, although for a more rigorous decrease in treatment time, other interventions need to be considered.

## Role of the funding source

The MOMENTUM Study is financially supported by Elekta and through in-kind contributions from the local institution. They did not have any role in the analysis, interpretation of data and writing or submission of this report.
