# Supplementary material for: Treatment time and learning curve analysis of 1.5 T MR-Linac workflows led by radiation oncologists or therapists
Source: Clin Transl Radiat Oncol. 2024 Dec 14;51:100901. doi: 10.1016/j.ctro.2024.100901 (PMC11759539; doi:10.1016/j.ctro.2024.100901)
Supplement: Supplementary Data 1 [file mmc1.docx]

**Supplemental figures and tables**

Table of content

[Supplemental figure 1. Flowchart of education structure for MR-Linac specialized RTT in the University Medical Center Utrecht. 2](#_Toc180441410)

[Supplemental figure 2. Flowchart of included patients 3](#_Toc180441411)

[Supplemental figure 3. Trends in total treatment time and online adaptation time. 4](#_Toc180441412)

[Supplemental figure 4. CUSUM analysis to determine a learning curve of RTT-led treatment fractions based on all available data. 6](#_Toc180441413)

[Supplemental figure 5. Duration of radiotherapy delivery for rectal cancer over time. 7](#_Toc180441414)

# Supplemental figure 1. Flowchart of education structure for MR-Linac specialized RTT in the University Medical Center Utrecht.

**Profile A: Clinical image processing**Previous experience as general RTT in clinical image processing required (>12 months)
Training in:

- Image registration: planning MR, MR external hospital, PET-CT
- Delineation: brain, head & neck, thorax, pelvis (CT&MR)

**Profile B: Treatment planning**Previous experience as general RTT in treatment planning required (>9 months)
Training in:

- Workshop Monaco & low complexity VMAT plans
- Workshop Monaco MRL Offline & low complexity MRL pre-treatment plans
- Experience in all low complexity plans, start higher complexity plans and start planning online on MRL under supervision
- Focus on high complexity plans and experience in online MRL workflow

**Hands-on training in house (duration based on personal skills and progress)**Training based on the “see one, do one, teach one” principle

- Workflow training for every new treatment site
- 1 week training on MR-simulation
- Sub-specialization of choice in profile A or profile B (see below)

**Theoretical MR-guided radiotherapy training (10 days) or in house training**Topics of training:

- Basic MR physics
- Positioning and tools
- Image registration and pitfalls (rigid and deformable)
- Geometric distortions
- MR safety
- MR only workflow
- Adaptive workflow
- MR-Linac logistics
- Motion management
- Patient, imaging and machine quality assurance (QA)

*Source: www.fontys.nl*

**Experience as general RTT working with the conebeam CT-Linac (minimal 1y)**With experience in treatment planning or clinical image processing

tra

**Bachelor Medical imaging and Radiation Therapy (4y) or in house education (3y)**Training in the operation of medical equipment in the field of radiology, radiotherapy, and nuclear medicine, aiming to prepare, conduct and process imaging or treatment. Training in the physiology of the human body, understanding which examinations are appropriate for specific medical conditions, the equipment required and how to operate the devices.
*Source: www.fontys.nl*

# Supplemental figure 2. Flowchart of included patients


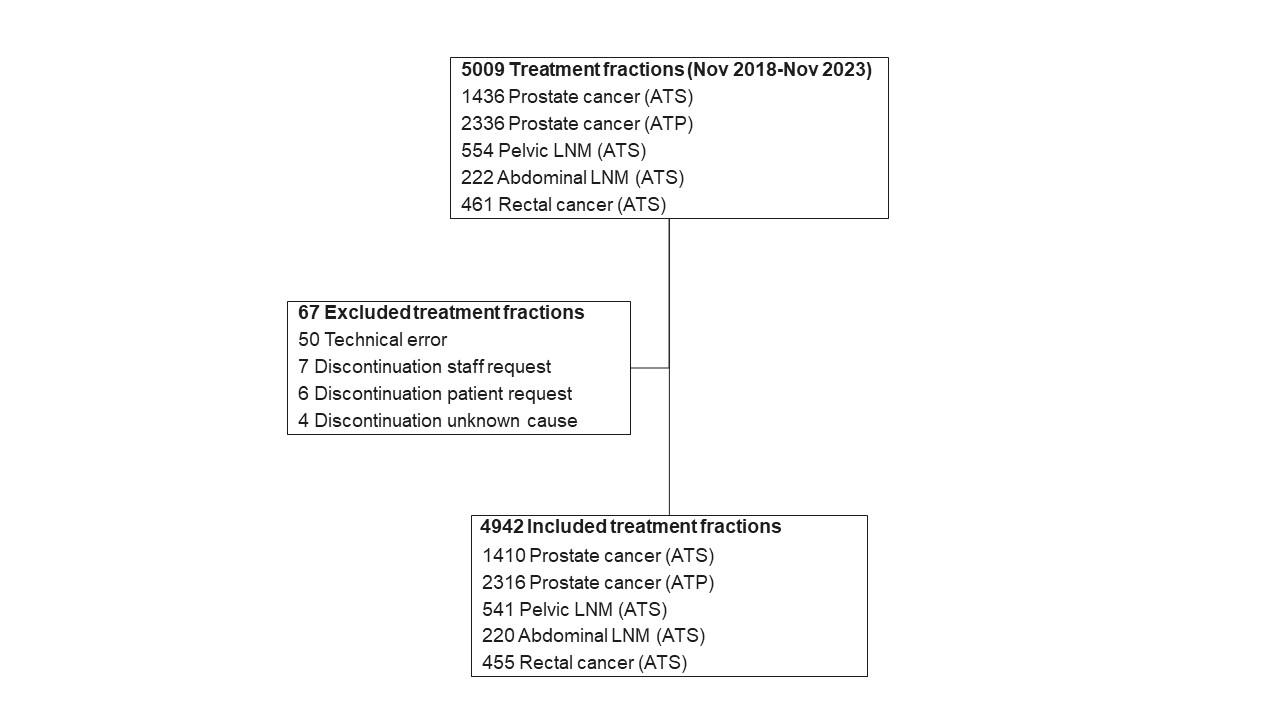


Abbreviations; ATS; Adapt-to-Shape; ATP, Adapt-to-Position; LNM, lymph node metastasis

# Supplemental figure 3. Trends in total treatment time and online adaptation time.

RO-led treatments are depicted in orange, and RTT-led treatment in blue. The trend in red was estimated based on a loess regression. Plots have different scales to improve readability.

Abbreviations; ATS, Adapt-to-Shape; ATP, Adapt-to-Position; LNM, lymph node metastasis; min, minutes; RO, radiation oncologist; RTT, radiation therapist

Continued; Supplemental figure 3. Trends in total treatment time and online adaptation time.
RO-led treatments are depicted in orange, and RTT-led treatment in blue. The trend in red was estimated based on a loess regression. Plots have different scales to improve readability.

Abbreviations; ATS, Adapt-to-Shape; LNM, lymph node metastasis; min, minutes; RO, radiation oncologist; RTT, radiation therapist

# Supplemental figure 4. CUSUM analysis to determine a learning curve of RTT-led treatment fractions based on all available data.

Abbreviations; CUSUM, cumulative sum; RTT, radiation therapist; ATS, Adapt-to-Shape; ATP, Adapt-to-Position; LNM, lymph node metastasis

Supplemental figure 5. Duration of radiotherapy delivery for rectal cancer over time.
RO-led treatments are depicted in orange, and RTT-led treatment in blue. The trend in red was estimated based on a loess regression.

Treatment fraction

Abbreviations; RT, radiotherapy; min, minutes; RO, radiation oncologist; RTT, radiation therapist
